# Takayasu’s Arteritis Diagnosed in an Adolescent Patient with Crohn’s Disease: Management of Biologicals

**DOI:** 10.3390/life11101019

**Published:** 2021-09-28

**Authors:** Kazuya Kishimoto, Yuji Nozaki, Toshiharu Sakurai, Koji Kinoshita, Masanori Funauchi, Itaru Matsumura

**Affiliations:** 1 Department of Hematology and Rheumatology, Faculty of Medicine, Kindai University, Osaka-Sayama, Osaka 589-8511, Japan; kazuya-k@med.kindai.ac.jp (K.K.); kkino@med.kindai.ac.jp (K.K.); mn-funa@med.kindai.ac.jp (M.F.); imatsumura@med.kindai.ac.jp (I.M.); 2 Department of Gastroenterology and Hepatology, Faculty of Medicine, Kindai University, Osaka-Sayama, Osaka 589-8511, Japan; sakurai@med.kindai.ac.jp

**Keywords:** Crohn’s disease, Takayasu’s arteritis, anti-TNFα monoclonal antibody, anti-IL-6 receptor antibody

## Abstract

We report a 14-year-old man with Crohn’s disease (CD) who developed right upper arm pain while being treated with the anti-tumor necrosis factor (TNF)-alpha monoclonal antibody, infliximab. There were no symptoms suggestive of active CD, but the inflammatory response was high, and a contrast-enhanced CT showed the occlusion of the right brachial artery. We diagnosed the patient as having Takayasu’s arteritis (TA) and started treatment with corticosteroids, then tapered off the steroids as the symptoms of TA resolved. Later, TA flared up, and his treatment was changed from infliximab to an anti-IL-6 receptor antibody, tocilizumab. The change to TCZ stabilized TA, but exacerbated CD. It is difficult to control both diseases at the same time, and the choice of biologics for treatment must be carefully considered.

## 1. Introduction

Crohn’s disease (CD) is an idiopathic chronic inflammatory bowel disease (IBD) and a relapsing systemic inflammatory disease affecting the gastrointestinal tract, particularly the terminal ileum and colon. CD is characterized pathologically by granulomatous fibrosing inflammation, resulting in significant complications, such as abscesses, fistulas, and strictures. Clinical features include abdominal pain, diarrhea, fever, and weight loss, often associated with extra-intestinal complications, involving organs such as the skin, eyes, joints, and cardiovascular system [1]. Takayasu’s arteritis (TA) is a chronic vasculitis of the aorta and its major branches. TA is characterized pathologically by a granulomatous inflammation similar to CD. The inflammation of the involved arteries can lead to stenosis, occlusions, dilatations, and/or aneurysms [2]. Clinical features range from an asymptomatic disease discovered as absent limb pulses to nonspecifc features of fever, malaise, weight loss, arthralgia, myalgia, and anemia to organ-specifc ischemic phenomena [3]. TA is a known extraintestinal complication associated with inflammatory bowel disease, and its complication with CD was first reported in 1976 [4]. Since then, several case reports have noted TA and CD in the same patient, though such coexistence has been theoretically estimated to occur in only 1 in 10 million individuals [5]. It has been suggested that there is a pathophysiological association between these two diseases. Inflammatory cytokines, such as the tumor necrosis factor (TNF)-α, are considered to play an important role in the pathogenesis of TA as well as CD [6,7]. Furthermore, polymorphisms in the IL-2, IL-6, and IL-12 genes may contribute to the susceptibility and pathogenesis of TA by altering the production of cytokines and inducing inflammation [8]. Inflammatory cytokines other than TNF-α also play an important role in the pathogenesis of CD. For example, IL-1β, IL-6, IL-8, and IL-16 contribute directly or indirectly to tissue damage by promoting the production of matrix metalloproteinases and growth factors, causing mucosal repair as well as ulceration [7]. Immunosuppressive therapy is the mainstay of treatment for these two diseases. The anti-TNF-α therapy has been reported to show beneficial therapeutic effects in both diseases [9,10,11,12]. Recently, the anti-IL-6 receptor antibody, tocilizumab (TCZ) is considered the effective therapeutic option in TA [13,14]. However, the efficacy of TCZ for CD has only been shown in a pilot study [15]. Herein, we report a case of a teenager who developed TA a few months after starting infliximab (IFX) for CD and was switched to TCZ due to the inadequate control of TA.

## 2. Case Presentation

**Figure 1:**
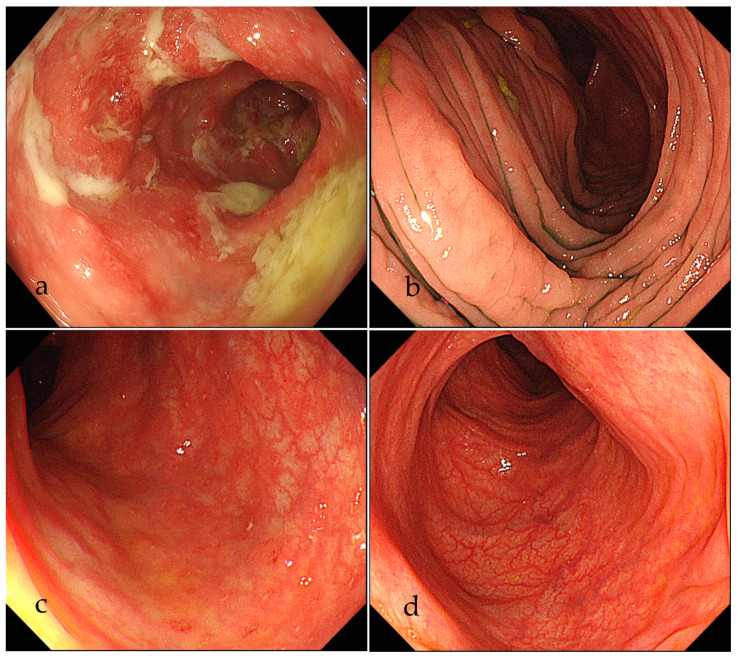
Colonoscopy findings at each stage. (**a**) extensive small erosions at the time of diagnosis of Crohn’s disease. (**b**) colonic mucosa with no activity before administration of tocilizumab. (**c**) multiple small erosions after administration of tocilizumab. (**d**) healed colonic mucosa after re-administration of infliximab.

**Figure 2:**
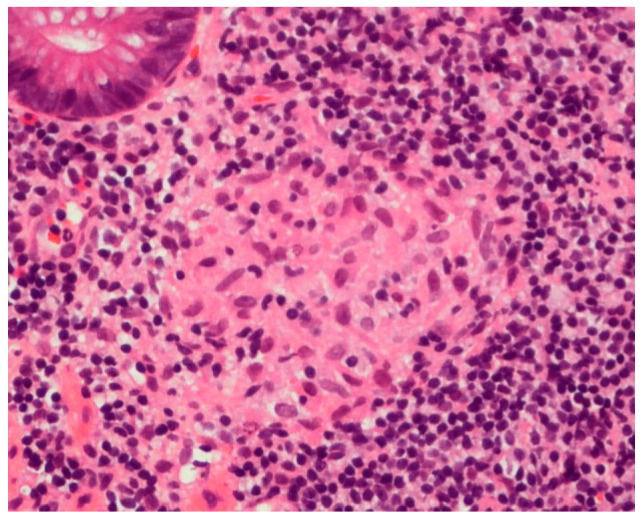
Non-caseating epithelioid granuloma in the colonic mucosa at the time of diagnosis of Crohn’s disease.

A 13-year-old man was admitted to our hospital because of fever, abdominal pain, and soft stool. A colonoscopy was performed and showed extensive small erosions from the ileum to the rectum (Figure 1a). A pathological examination showed non-caseating epithelioid granuloma (Figure 2). He was diagnosed with CD of the ileo-colic type. Hence, a therapy with 5-aminosalicylic acid (5-ASA) and IFX was started, and CD was well controlled.

**Figure 3:**
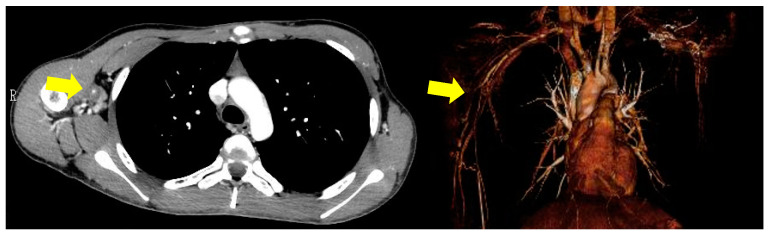
Contrast-enhanced CT images at the time of diagnosis of Takayasu’s disease. The yellow arrow showed a narrowing of the brachial artery.

**Figure 4:**
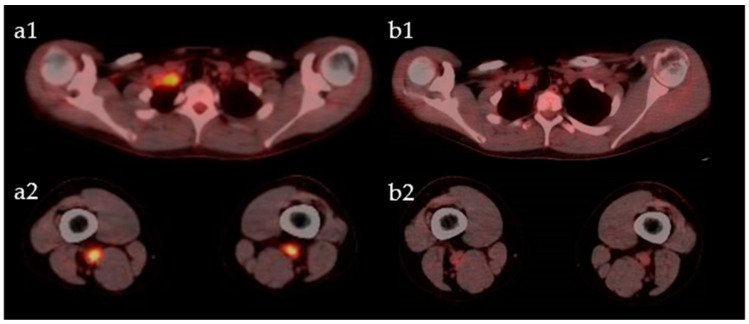
FDG-PET images. FDG accumulation in right subclavian artery (**a1**) and bilateral femoral artery (**a2**) before administration of tocilizumab. FDG accumulation in right subclavian artery (**b1**) and bilateral femoral artery (**b2**) a few months after discontinuation of tocilizumab. SUVs are improving, indicating that Takayasu’s arteritis is little activity.

Six months later, he was admitted to our hospital for scrutiny due to fever and pain in the right arm. Blood pressure was 114/65 mmHg and 96/49 mmHg in the left and right brachial arteries, respectively. His body temperature was 37.5 °C. Laboratory results showed an increase in acute phase indicators (erythrocyte sedimentation rate: 61 mm/h, C-reactive protein: 8.98 mg/dL), and the results of autoimmune markers were within normal ranges. A contrast-enhanced CT showed a narrowing of the lumen from the axillary artery to the brachial artery (Figure 3), which satisfied 4 out of 6 items in the ACR classification criteria (age at disease onset ≤40 years; claudication of extremities; blood pressure difference >10 mmHg; arteriogram abnormality) [16]. Therefore, we diagnosed TA and started oral steroid treatment (prednisolone: PSL 30 mg/day). We added azathioprine and reduced the dose of PSL to 10 mg. Both TA and CD were stable for a while, but CRP gradually increased, and the pain in the right arm intensified. A contrast-enhanced CT showed a further narrowing of the lumen of the right axillary artery, and a few months later the patient became aware of a pain in both thighs. Therefore, we performed a fluoro-D-glucose-positron emission tomography (FDG-PET) to evaluate the systemic activity of TA. FDG-PET images showed an accumulation of FDG in the right subclavian artery, right axillary artery to right brachial artery, bilateral femoral artery, and the origin of the ascending aorta, and the SUVs were 5.7, 2.1, 8.7, and 2.8, respectively (Figure 4a). At that time, there was no evidence of CD activity on the colonoscopy (Figure 1b). Considering these results, TA was clearly exacerbated, and the treatment had to be reconsidered. So, we decided to switch from IFX to a subcutaneous injection of TCZ 162 mg weekly and increased the dose of PSL to 15mg. The change to TCZ improved the symptoms of TA and made it possible to reduce the dose of steroids.

However, the abdominal pain increased 5 months after changing to TCZ. A colonoscopy showed multiple small erosions at the end of the ileum and small erosions with redness from the cecum to the rectum (Figure 1c). So, we decided to use IFX again and changed from TCZ to IFX 5mg/kg every 4 weeks. At the same time, the dose of PSL was increased to 20 mg. FDG-PET images taken a few months after re-administration of IFX showed little activity (Figure 4b), and the subjective symptoms were stable. About 1 year after re-administering IFX, a colonoscopy revealed that the mucosa was in a healed state (Figure 1d). Currently, a treatment with PSL 10 mg, 5-ASA and IFX 5mg/kg every 4 weeks is ongoing.

## 3. Discussion

TA is a large, non-specific vasculitis of unknown origin that causes inflammatory wall thickening of the aorta and its major branches, pulmonary arteries, and coronary arteries, resulting in stenosis, occlusion, or dilated lesions. An ischemic injury specific to the dominant organ of the stenotic or occluded artery, or an aneurysm due to dilated lesion, is the main clinical pathology. The clinical manifestations vary depending on the vascular territory in which the lesion occurs, resulting in a wide variety of clinical symptoms [2,3]. Corticosteroids are used as the first line of treatment [17], and many cases go into remission but often relapse [18]. When relapses occur, immunosuppressive agents such as methotrexate [19], azathioprine [20], and cyclophosphamide [21] are used in addition to increasing the dose of steroids, but there are many cases that are refractory to these drugs, and the treatment of such refractory TA is important. In 2004, Hoffman et al. investigated the efficacy of TNF-α inhibitor therapy with IFX and etanercept (ETN) in patients with refractory TA who had difficulty achieving remission despite a high-dose steroid therapy and who had repeated relapses. As much as 66.7% of patients achieved remission, and the latter was maintained for 1–3.3 years after the discontinuation of steroids [9]. In 2012, a multicenter clinical trial was conducted in France to evaluate the efficacy of IFX in patients with refractory TA and showed that IFX was effective in reducing steroids and inducing remission [22]. Recently, anti-IL-6 receptor antibody therapy has also been shown to be effective. IL-6 is one of the pro-inflammatory cytokines that plays an important role in various inflammatory and autoimmune diseases. In the serum of patients with TA, IL-6 levels were reported to increase in correlation with the disease activity, suggesting the importance of IL-6 in TA [23]. In 2018, the TAKT study suggested the superiority of tocilizumab over the placebo for the recurrence of TA, with no new safety concerns, establishing a treatment for refractory TA [24].

CD, an inflammatory bowel disease of unknown origin, consists of granulomatous inflammatory lesions with ulceration and fibrosis that can occur in any part of the gastrointestinal tract. It can also involve areas other than the gastrointestinal tract, and the clinical feature varies depending on the location and extent of the lesions. Systemic symptoms such as fever, nutritional disorders, anemia, and systemic complications such as arthritis, iritis, and liver damage may occur. The treatment of CD is mainly pharmacological, using 5-ASA, steroids, immunosuppressive agents, and biologicals to reduce inflammation and excessive immune effects [1]. The early introduction of the anti-TNF-α antibody after diagnosis has been shown to improve long-term prognosis, and some reports suggest that an early introduction should be considered [25], especially in younger patients, patients with extensive disease at diagnosis, patients with severe rectal or anal disease, and patients requiring steroids at diagnosis [26].

In the present case, the patient was young and had an extensive disease at the time of diagnosis, so an anti-TNF-α antibody therapy, IFX, was introduced shortly after the diagnosis. After the start of treatment, CD remained in clinical remission, but within a year fever and a pain in the right upper arm were observed, and a contrast-enhanced CT showed that the patient had TA. The prevalence of CD in patients with TAK is rare and thought to be 0.05 to 0.2% [27]. The mechanism of CD and TA complications has not been elucidated, and it has only been speculated that some common immunohistological features may be involved in the complications of both diseases. Both diseases are histopathologically characterized by granulomatous inflammation, and inflammatory cytokines such as TNF-α, IL-6, IL-8, IL-12, and IL-18 are involved in their pathogenesis [7,28]. Anti-TNF-α antibody preparations are effective for CD, but it has also been pointed out that it may be associated with the risk of developing vasculitis, including TA [29].

In this case, after the diagnosis of TA, the patient was started on the recommended first-line steroid: PSL 30 mg. CD was maintained in clinical remission with 5-ASA and IFX, the symptoms of TA disappeared with oral steroids, and there was no inflammatory reaction. However, when PSL was reduced to 10 mg, the pain in the right upper arm increased, and a new pain in both thighs, which was not present at the time of diagnosis, was noted. If TA flares up, the patient needs to be given a higher dose of steroids [17], but the adolescent boy was very anxious about the side effects of increasing the dose of steroids, especially the appearance of a moon face, and for this reason we could not get his consent to increase the steroid dose sufficiently. Although the patient was treated symptomatically for a while, the CRP increased to 9.21 mg/dL, and the FDG-PET showed a worsening vasculitis activity. The intensified treatment for TA was therefore inevitable. The goal of the medical treatment of TA is to control vascular inflammation and avoid irreversible vascular damage that can lead to stenosis and aneurysms, for which the anti-inflammatory effects of steroids are necessary [18]. However, in this case, we were unable to increase the dose of steroids sufficiently, so we had to rely on biological agents for the treatment of TA.

Since the efficacy of IFX is not likely to improve, we considered changing to TCZ, and although the efficacy of TCZ for CD had been reported only in the Piot study [15] and there was not sufficient evidence, the decision to change to TCZ was made after a thorough consultation with the patient and his parents. After switching to TCZ, the symptoms of TA disappeared and no inflammatory reaction was observed. Therefore, PSL was gradually reduced. However, six months later, abdominal pain was observed, and a colonoscopy showed a flare-up of CD.

IL-6 is involved in the protection of intestinal epithelial cells. In vitro, IL-6 induces anti-apoptotic proteins in colon epithelial cells against apoptosis [30]. Further, IL-6 function is required to stimulate epithelial proliferation during intestinal inflammation, and thus IL-6 may also play a beneficial role in wound repair in the intestine early after injury [31]. In this case, we speculate that the use of the anti-IL-6R antibody preparation inhibited the meaningful role of IL-6 in the intestinal tract and worsened CD.

It may be difficult to control both diseases at the same time. In this case, the change from IFX to TCZ resulted in an improvement of TA but a relapse of CD. The subsequent choice of treatment was troubling, and we opted for the re-administration of IFX to control CD. It was expected that this would cause TA to flare up, so the dose of PSL was increased to 20 mg after a thorough consultation. The progress of both diseases has been good since then, and the steroids are being gradually reduced.

In the future, TA may flare up, and it is expected that it will be even more difficult to select a treatment method in that case. I consider that tofacitinib (TOF), a Janus kinase inhibitor indicated for the treatment of IBD, is one of the treatments available for that case. TOF acts as a multi-cytokine blocker by inhibiting the enzyme, and Kuwabara et al. have demonstrated, in 2020, its efficacy for TA complicated by ulcerative colitis resistant to both TNF-α and IL-6 inhibitors [32]. The other option is ustekinumab, which is an IL-12/23p40 monoclonal antibody indicated for the treatment of CD, and was reported to be effective in the treatment of TA by Terao et al. in 2016 [33]. It is hoped that both drugs will become available for use in TA through future clinical trials.

## 4. Conclusions

The development of TA during the treatment of CD is rarely observed. Biologicals are effective in both diseases, but we should be very careful in the choice.

## Data Availability

No applicable.
